# The Iterative Learning Gain That Optimizes Real-Time Torque Tracking for Ankle Exoskeletons in Human Walking Under Gait Variations

**DOI:** 10.3389/fnbot.2021.653409

**Published:** 2021-05-28

**Authors:** Juanjuan Zhang, Steven H. Collins

**Affiliations:** ^1^Department of Mechanical Engineering, Carneigie Mellon University, Pittsburgh, PA, United States; ^2^College of Artificial Intelligence, Nankai University, Tianjin, China; ^3^Department of Mechanical Engineering, Stanford University, Stanford, CA, United States

**Keywords:** exoskeleton, iterative learning, control, rehabilitation, gait assistance

## Abstract

Lower-limb exoskeletons often use torque control to manipulate energy flow and ensure human safety. The accuracy of the applied torque greatly affects how well the motion is assisted and therefore improving it is always of interest. Feed-forward iterative learning, which is similar to predictive stride-wise integral control, has proven an effective compensation to feedback control for torque tracking in exoskeletons with complicated dynamics during human walking. Although the intention of iterative learning was initially to benefit average tracking performance over multiple strides, we found that, after proper gain tuning, it can also help improve real-time torque tracking. We used theoretical analysis to predict an optimal iterative-learning gain as the inverse of the passive actuator stiffness. Walking experiments resulted in an optimum gain equal to 0.99 ± 0.38 times the predicted value, confirming our hypothesis. The results of this study provide guidance for the design of torque controllers in robotic legged locomotion systems and will help improve the performance of robots that assist gait.

## 1. Introduction

Being able to reduce interface impedance, increase the reactiveness of robotic systems and thus improve human safety and comfort (Haddadin et al., 2008; Lasota et al., 2014), torque control has been widely used in physical human-robot interactive systems. This is especially true in lower-limb systems, which help human bodies to locomote and were involved in high density of energy exchange. Torque control enables easy manipulation of energy flow from the robot to the human, which is one major research interest in the field of biomechanics (Veneman et al., 2007; Sawicki and Ferris, 2009; Stienen et al., 2010; Malcolm et al., 2013; Jackson and Collins, 2015). It has also been used to exploit passive system dynamics or render virtual systems with different dynamics in humanoid robots (Pratt et al., 1997), robot prostheses (Sup et al., 2009; Caputo and Collins, 2013), and exoskeletons (Kawamoto et al., 2010; Witte et al., 2015). In torque controlled human-robot interactive systems, torque tracking accuracy equals precision of the applied intervention or assistance and thus directly affects how well the assisted motion is. Therefore, improving torque control performance has always been an active interest in the field of lower-limb human-robot interactive systems.

Due to the presence of complicated, time-varying, and highly non-linear human dynamics, interaction dynamics and transmission dynamics, a fixed accurate system model was neither easy to get nor very meaningful due to the fast changes of system mechanical properties when human walk. Thus, high accuracy torque tracking of lower-limb exoskeletons was not easy to achieve. Different control methods have been introduced to improve torque tracking performances of lower-limb wearable robotic devices (van Dijk et al., 2013; Zanotto et al., 2013; Zhang et al., 2015, 2017a,b; Zhang and Collins, 2017). Among them, the combination of model-free, integration-free feedback control and iterative learning showed high accuracy and has been applied in multiple robotic legged locomotion systems (van Dijk et al., 2013; Zhang et al., 2015, 2017a,b; Zhang and Collins, 2017). This control architecture ignored the complicated and changing system dynamics caused by human-robot interactions and transmission frictions. It focused on the power transmission subsystem which was modeled as a linear spring. A P-type learning term (Arimoto et al., 1984) serves as a stride-wise integral control entity and reduces steady-state errors by exploiting the cyclic behavior of walking. Therefore, the structure is analogous to a traditional PID controller, in which tracking capability, stability and steady-state error manipulation are all managed. In this structure, iterative learning term is added due to the cyclic behavior of walking and is used in a feed-forward way, it thus has lagged response to real-time torque tracking errors. Therefore, the addition of this term in the control structure was expected to eliminate errors nominal to a stabilized gait pattern. However, walking experiments showed interactions of iterative learning gain with real-time tracking performance after stabilization of the learning process. This suggested a possibility to further improve real-time torque tracking performance in lower-limb exoskeletons.

Since the proposal of the basic iterative learning control concept in the 1980s (Arimoto et al., 1984), various techniques have been developed to optimize the learning gain. One approach was to enforce system convergence to follow some gradient of an objective function defined by the quadratic cost of tracking errors (Togai and Yamano, 1985; Moore, 1993; Fukuda and Shin, 1998), or a weighted combination of tracking errors and change in control inputs (Amann et al., 1996). Other works defined learning gains by maximizing convergence speed of control inputs (Atkeson and Mclntyre, 1986; Hać, 1990; Heinzinger et al., 1992; Saab et al., 1997). These early algorithms dealt with invariant and deterministic system dynamics. More recent work has discussed algorithms to compute optimal and sub-optimal iterative learning gains under measurement noises for time-varying linear (Saab, 2003) and non-linear systems (Saab, 2005).

These existing works mainly optimized learning gains by expediting the convergence process of learning. In addition, fairly good knowledge of the system dynamics and noise level were available. However, in the problem of exoskeleton assisted walking, there exist stride-to-stride gait variations and gait adaptation, which make walking not exactly periodical. This results in tracking errors even after stabilization of the iterative learning process. The control architecture combining feedback and iterative learning depends mainly on the feedback part to contain these errors, which might be further reduced by tuning the gain of the iterative learning part.

This paper explores the possibility of optimizing the post-stabilization real-time control performance of Arimoto's P-type learning control on lower-limb legged robots driven by series elastic actuators. Given complicated, varying and uncertain human-robot interaction, transmission friction dynamics, stride-to-stride variations of human gait, and limited knowledge of gait variation distribution, this study investigated whether and how one could maximize the real-time torque control performance. Previous studies have shown that the torque tracking performance can be achieved by a proper feedback + iterative learning structure (Zhang et al., 2015, 2017a), and optimized passive stiffness values of series elastic actuators (Zhang and Collins, 2017). On top of those, this study aims to further improve torque tracking performance of control architecture in Zhang et al. (2015, 2017a,b), and Zhang and Collins (2017) in lower-limb exoskeletons and other robotic locomotion systems. In particular, we look into the possibility of relating the optimal learning gain based on real-time tracking errors with actuator passive stiffness and desired quasi-stiffness. Results of this study are expected to follow previous work (Zhang et al., 2015, 2017a; Zhang and Collins, 2017) to further improve assisted torque assertion accuracy for lower-limb exoskeletons and prosthesis, and thus the locomotion performance of the resulting coupled human-robot systems.

## 2. Methods

We investigated the effects of iterative learning gain on the torque tracking performance of lower-limb exoskeletons using an ankle exoskeleton driven by a uni-directional Bowden cable.

We hypothesized an optimal value of iterative learning gain based on theoretical analysis and tested it during exoskeleton assisted walking experiments.

In testing the hypothesis, multiple desired quasi-stiffness values, i.e., torque vs. ankle angle relationships, were implemented, each tested with multiple actuator passive stiffness values. For each of the desired stiffness and passive stiffness combination, multiple iterative learning gains were tested. Every experiment session identified by a unique set of {Learning Gain, Desired Stiffness, Passive Stiffness} values required the participant to walk on the treadmill with a fixed speed for at least one hundred strides after stabilization of the iterative learning process. The existence and value of the learning gain were then investigated by comparing the torque tracking errors of different experiment sessions.

### 2.1. Exoskeleton System and Simplified Model

**Figure 1:**
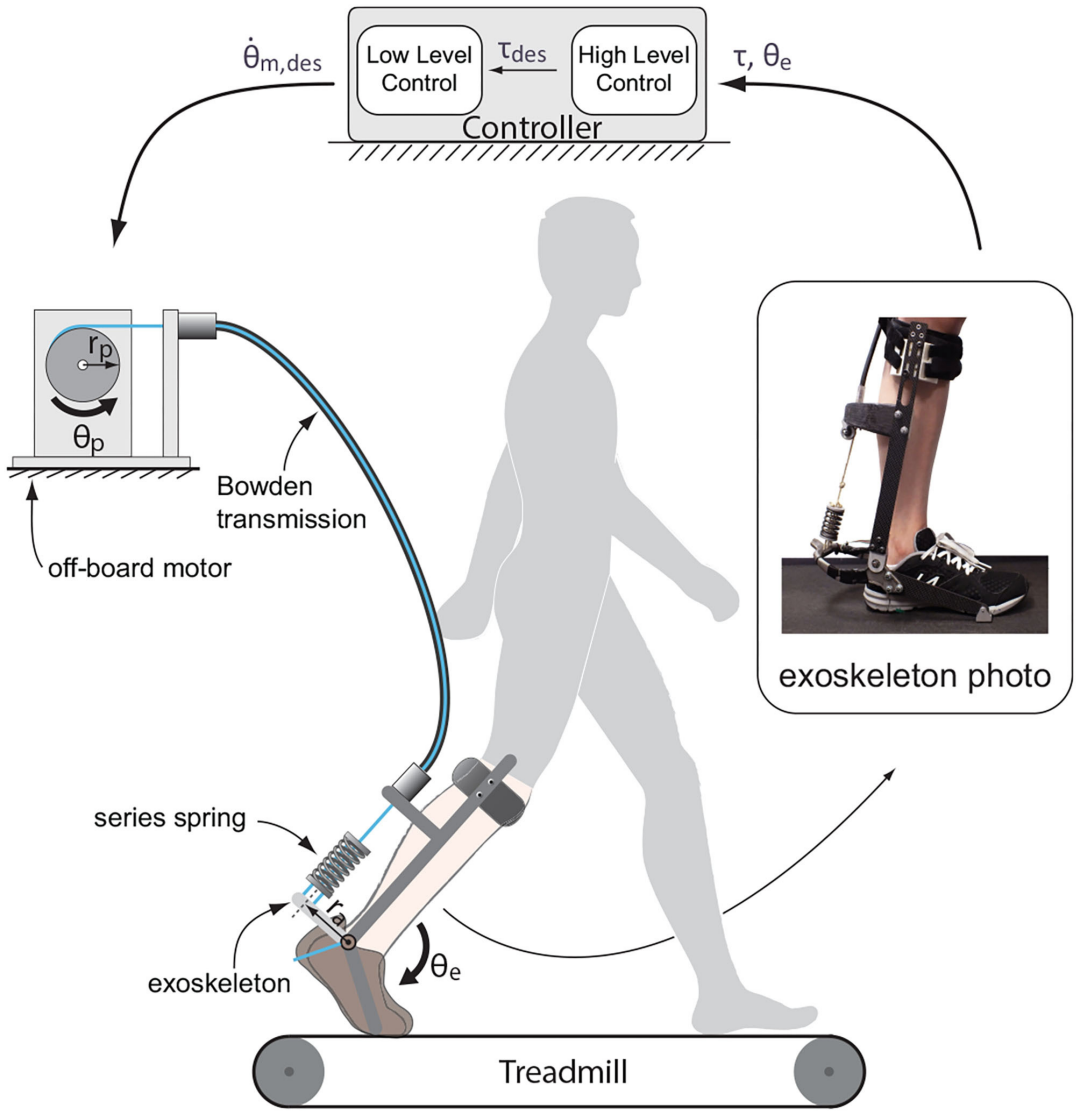
Tethered ankle exoskeleton system. Besides sensor data acquisition and outputting control signal to the motor, the dedicated controller has two main computational modules: a high-level controller that generates the desired torque according to ankle angle in real-time, and a low-level controller that generates control signal as desired motor velocity according to desired torque, applied torque and current motor status. Changes in motor motion status tunes the torque applied through Bowden cable transmission to the end effector, an ankle exoskeleton with series spring.

The system we investigated was a tethered ankle exoskeleton made of an off-board real-time controller and geared motor, a uni-directional Bowden cable transmission with a series spring, and an exoskeleton frame that interfaced with the human body (Figure 1).

A real-time control unit (ACE1103, dSPACE Inc.) was used to sample sensory data at 5 kHz, filter them at 200 Hz, and then compute control outputs in terms of desired motor velocity. The actuation unit included a low-inertia 1.6 kW AC servo motor, a 5:1 gear, and a motor driver configured to velocity control mode (BSM90N-175AD, GBSM90-MRP120-5, and MFE460A010B, Baldor Electric Co.). A digital encoder (E4, US Digital Corp.) was used to measure motor position.

A uni-directional Bowden cable was used to transmit forces from the motor side to the exoskeleton side. The cable was made of a coiled-steel conduit (415310-00, Lexco Cable Mfg.) and a 0.003 m diameter synthetic rope. A spring (DWC-148M-12, Diamond Wire Spring Co.) of a 190 N·m·rad^−1^ effective stiffness (in terms of ankle position) was attached at the end of the rope to realize series elastic actuation.

The exoskeleton frame applied an plantarflflexion torque to human ankle. Torque was measured using strain gauges (MMF003129, Micro-Measurements), and amplified using a 1 kHz signal conditioner (CSG110, Futek Inc.). Ankle angle was measured using a digital encoder (E5, US Digital Corp.).

We made the following assumptions in generating a simplified model of the system for the purpose of theoretical analysis:

1. Both static and dynamic frictions in Bowden cable transmission were zero. Thus, cable tension at the motor output pulley side was always the same as the exoskeletons side. This tension was denoted as *F*.
2. The Bowden cable transmission and the series spring together behaved as a linear spring, i.e.,
  (1) $$\begin{array}{l} F=K_{c}\cdot \left(r_{p}\cdot \theta_{p}-r_{a}\cdot \theta_{e}\right) \end{array}$$
  where *K*_*c*_ was the combined effective stiffness of the Bowden cable and series spring; θ_*p*_ and θ_*e*_ were the pulley and exoskeleton joint positions relative to where the Bowden cable first started to go slack; *r*_*p*_ and *r*_*a*_ were the pulley radius and the lever arm at the ankle joint.
3. Exoskeleton joint rotated in a small range. Thus, the cable tension lever arm at exoskeleton joint side was almost constant, i.e., *r*_*a*_ was fixed. Therefore, torque applied to human body by the exoskeleton was always
  (2) $$\begin{array}{l} \tau =F\cdot r_{a}. \end{array}$$

Denoting the aspect ratio of transmission as

(3) $$\begin{array}{l} R=\frac{r_{p}}{r_{a}} \end{array}$$

and combining it with Equations (1) and (2), the torque applied by the exoskeleton to the human ankle was

(4) $$\begin{array}{l} \tau =F\cdot r_{a}\\ \;=r_{a}^{2}\cdot K_{c}\left[\theta_{p}\frac{r_{p}}{r_{a}}-\theta_{e}\right]\\ \;=K_{t}\left(\theta_{p}R-\theta_{e}\right) \end{array}$$

Where *K*_*t*_ was the transmission stiffness which related applied torque to exoskeleton joint. It was defined as

(5) $$\begin{array}{l} K_{t}=r_{a}^{2}\cdot K_{c}. \end{array}$$

### 2.2. Controllers

#### 2.2.1. Low Level Control

Prior work in the field found that in real-time torque tracking of lower-lime exoskeletons during walking, the combination of model-free, integral-action-free feedback control and iterative learning were most effective controller with complicated and time-varying dynamics of human-robot interactive systems (Zhang et al., 2015, 2017a,b; Zhang and Collins, 2017). The overall torque tracking performance of Equation (6) is a combined effect of feedback control and iterative learning. To simplify theoretical analysis and experimental tests of the existence of the optimal learning gains, only iterative learning was used as the lower level controller in this study. The controller was expressed as:

(6) $$\begin{array}{l} \theta_{p,des}\left(i,n+1\right)=\theta_{p,des}\left(i,n\right)-K_{l}\cdot e_{\tau }\left(i,n\right)\\ \;{\dot{\theta }}_{p,des}\left(i,n\right)=\frac{1}{T}\left(\theta_{p,des}\left(i,n\right)-\theta_{p}\right)\\ \;{\dot{\theta }}_{m,des}\left(i,n\right)=\frac{N}{T}\left(\theta_{p,des}\left(i,n\right)-\theta_{p}\right) \end{array}$$

Here *i* is the time index or number of control cycles elapsed within the current stride. *n* denotes this stride and *n* + 1 is the next. τ, τ_*des*_ and *e*_τ_ = τ − τ_*des*_ are the measured exoskeleton torque, the desired torque and the torque error, respectively. *K*_*l*_ is the iterative learning gain. The motor run in velocity mode. The desire motor output pulley velocity ${\dot{\theta }}_{p,des}$ was always converted to desired motor velocity ${\dot{\theta }}_{m,des}$ before commanded. *T* is a constant simulating the rise time of motor position tracking and *N* is the gear ratio. As shown by the equation, torque error in the current stride will not affect the control input until the next stride. Therefore, iterative learning was used in a feed-forward way.

#### 2.2.2. High Level Control: Linear Desired Quasi-Stiffness

**Figure 2:**
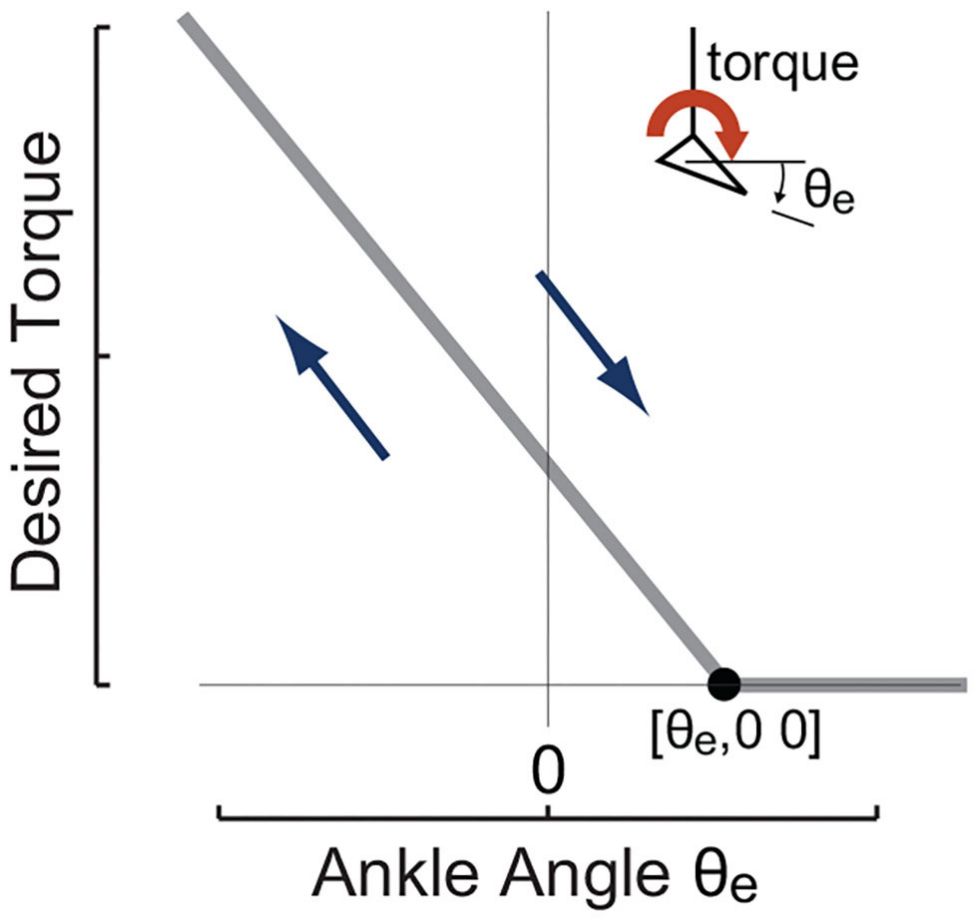
The ankle angle based high-level desired torque curve imposed in experiments to realize different desired quasi-stiffness profiles. It commands desired torque that is linearly proportional to exoskeleton joint angle θ_*e*_ defined by anchor point [θ_*e*,0_ 0] and desired quasi-stiffness *K*_*des*_.

The main high-level controller used in this study was a linear torque vs. ankle angle curve, i.e., a equilibrium-controlled stiffness as shown in Figure 2 and expressed in Equation (7).

(7) $$\begin{array}{l} \tau_{des}=-K_{des}\left(\theta_{e}-\theta_{e,0}\right)\\ \tau_{des}=\max\left(\tau_{des},0\right) \end{array}$$

where θ_*e*,0_ denotes the maximum ankle position to apply external force and *K*_*des*_ is a quasi-stiffness to be realized.

### 2.3. Theoretical Analysis on Optimal Iterative Learning Gain

In this analysis, we assumed perfect motor position tracking, i.e.,

(8) $$\begin{array}{l} \theta_{p}\left(i,n\right)\equiv \theta_{p,des}\left(i,n\right) \end{array}$$

for any index *i*. θ_*p*_ is the measured motor pulley position and θ_*p,des*_ is the desired one. *n* denotes the *n*th stride and *i* denotes the current index counted from the latest stride start time. The iterative learning of desired motor position was conducted as (Zhang et al., 2015, 2017a,b; Zhang and Collins, 2017),

(9) $$\begin{array}{l} \theta_{p,des}\left(i,n+1\right)=\theta_{p,des}\left(i,n\right)-K_{l}\cdot e_{\tau }\left(i,n\right) \end{array}$$

Torque transmission was modeled in Equation (4) and the desired torque was set as Equation (7).

In this study, we were interested in the real-time torque tracking performance under gait variations after the stabilization of iterative learning, i.e., disturbance rejection performance of the controller. Therefore, we assumed that at stride *n* − 1 and time index *i* the learning controller has reached stabilization with perfect torque tracking, i.e.,

(10) $$\begin{array}{l} \;\tau \left(i,n-1\right)=\tau_{des}\left(i,n-1\right)\\ e_{\tau }\left(i,n-1\right)=\tau \left(i,n-1\right)-\tau_{des}\left(i,n-1\right)=0. \end{array}$$

The dynamic changes of the desired and generated torque in the next strides due to human gait variations are then investigated hereinafter.

Assuming an ankle kinematics change from stride *n* − 1 to *n* at index *i* of

(11) $$\begin{array}{l} \Delta \theta_{e}\left(i,n\right)=\theta_{e}\left(i,n\right)-\theta_{e}\left(i,n-1\right), \end{array}$$

then the desired torque changes by

$$\begin{array}{c} \Delta \tau_{des}\left(i,n\right)=\tau_{des}\left(i,n\right)-\tau_{des}\left(i,n-1\right)\\ \;=-K_{des}\cdot \Delta \theta_{e}\left(i,n\right) \end{array}$$

Based on the iterative learning rule in Equation (9) and the assumption of perfect motor position tracking in Equation (8), current desired and actual motor position are

(12) $$\begin{array}{l} \theta_{p}\left(i,n-1\right)=\theta_{p,des}\left(i,n\right)\\ \;=\theta_{p,des}\left(i,n-1\right)-K_{l}\cdot e_{\tau }\left(i,n-1\right)\\ \;=\theta_{p,des}\left(i,n-1\right)-K_{l}\cdot 0\\ \;=\theta_{p,des}\left(i,n-1\right). \end{array}$$

Therefore, combining Equation (12) with Equation (4), the measured torque at stride *n* and index *i* is

(13) $$\begin{array}{l} \tau \left(i,n\right)=\tau \left(i,n-1\right)-K_{t}\cdot \Delta \theta_{e}\left(i,n\right) \end{array}$$

and the desired torque is

(14) $$\begin{array}{l} \tau_{des}\left(i,n\right)=\tau_{des}\left(i,n-1\right)+\Delta \tau_{des}\left(i,n\right)\\ \;=\tau_{des}\left(i,n-1\right)-K_{des}\cdot \Delta \theta_{e}\left(i,n\right) \end{array}$$

Combining Equations (20) and (21), for stride *n* and index *i*, the torque error is

(15) $$\begin{array}{l} e_{\tau }\left(i,n\right)\\ \begin{array}{c} =\tau \left(i,n\right)-\tau_{des}\left(i,n\right)\\ =\tau \left(i,n-1\right)-K_{t}\Delta \theta_{e}\left(i,n\right)-\left[\tau_{des}\left(i,n-1\right)-K_{des}\Delta \theta_{e}\left(i,n\right)\right]\\ =e_{\tau }\left(i,n-1\right)+\left(K_{des}-K_{t}\right)\Delta \theta_{e}\left(i,n\right)\\ =0+\left(K_{des}-K_{t}\right)\Delta \theta_{e}\left(i,n\right)\\ =\left(K_{des}-K_{t}\right)\Delta \theta_{e}\left(i,n\right) \end{array} \end{array}$$

This means with perfect torque tracking in stride *n* − 1, the torque error of stride *n* is minimized when the desired and passive stiffness match. This agrees with a previous study on the optimization of passive stiffness for torque tracking (Zhang and Collins, 2017). Next, at index *i* of stride *n* + 1, the desired/actual motor position (equal per perfect motor tracking assumption 8) is

(16) $$\begin{array}{l} \theta_{p,des}\left(i,n+1\right)=\theta_{p,des}\left(i,n\right)-K_{l}\cdot e_{\tau }\left(i,n\right)\\ \;=\theta_{p,des}\left(i,n\right)-K_{l}\cdot \left(K_{des}-K_{t}\right)\cdot \Delta \theta_{e}\left(i,n\right). \end{array}$$

In case there is no change in ankle kinematics from stride *n* to *n* + 1 at time index *i*, i.e., θ_*e*_(*i, n* + 1) = θ_*e*_(*i, n*), the desired torque values have τ_*des*_(*i, n* + 1) = τ_*des*_(*i, n*). The actual torque at current stride and index is

(17) $$\begin{array}{l} \tau \left(i,n+1\right)\\ \begin{array}{c} =K_{t}\left[\theta_{p}\left(i,n+1\right)R-\theta_{e}\left(i,n+1\right)\right]\\ =K_{t}\left[\left(\theta_{p,des}\left(i,n\right)-K_{l}\left(K_{des}-K_{t}\right)\Delta \theta_{e}\left(i,n\right)\right)R-\theta_{e}\left(i,n+1\right)\right]\\ =K_{t}\left[\left(\theta_{p,des}\left(i,n\right)-K_{l}\left(K_{des}-K_{t}\right)\Delta \theta_{e}\left(i,n\right)\right)R-\theta_{e}\left(i,n\right)\right]\\ =K_{t}\left[\theta_{p,des}\left(i,n\right)R-K_{l}\left(K_{des}-K_{t}\right)R\Delta \theta_{e}\left(i,n\right)-\theta_{e}\left(i,n\right)\right]\\ =\tau \left(i,n\right)-K_{t}K_{l}\left(K_{des}-K_{t}\right)R\Delta \theta_{e}\left(i,n\right). \end{array} \end{array}$$

Therefore, the latest torque error, i.e., that of stride *n* + 1 and index *i*, is

(18) $$\begin{array}{l} e_{\tau }\left(i,n+1\right)\\ \begin{array}{c} =\tau \left(i,n+1\right)-\tau_{des}\left(i,n+1\right)\\ =\tau \left(i,n+1\right)-\tau_{des}\left(i,n\right)\\ =\tau \left(i,n\right)-K_{t}K_{l}\left(K_{des}-K_{t}\right)R\Delta \theta_{e}\left(i,n\right)-\tau_{des}\left(i,n\right)\\ =\left(K_{des}-K_{t}\right)\Delta \theta_{e}\left(i,n\right)-K_{t}K_{l}\left(K_{des}-K_{t}\right)R\Delta \theta_{e}\left(i,n\right)\\ =\left(K_{des}-K_{t}\right)\left(1-K_{t}K_{l}R\right)\Delta \theta_{e}\left(i,n\right) \end{array} \end{array}$$

if *K*_*t*_·*K*_*l*_·*R* = 1, i.e., $K_{l}=\frac{1}{K_{t}R}$, we have

$$e_{\tau }\left(i,n+1\right)=0,$$

and balance is restored.

On the other hand, if there exists ankle kinematics change from stride *n* to *n* + 1 at index *i*, i.e.,

(19) $$\begin{array}{l} \Delta \theta_{e}\left(i,n+1\right)=\theta_{e}\left(i,n+1\right)-\theta_{e}\left(i,n\right)\neq 0, \end{array}$$

we have

(20) $$\begin{array}{l} \tau_{des}\left(i,n+1\right)=\tau_{des}\left(i,n\right)-K_{des}\cdot \Delta \theta_{e}\left(i,n+1\right), \end{array}$$

and the actual torque at (*i, n* + 1) is

(21) $$\begin{array}{l} \tau \left(i,n+1\right)\\ \begin{array}{c} =K_{t}\left[\theta_{p}\left(i,n+1\right)R-\theta_{e}\left(i,n+1\right)\right]\\ =\tau \left(i,n\right)-K_{t}K_{l}\left(K_{des}-K_{t}\right)R\Delta \theta_{e}\left(i,n\right)-K_{t}\Delta \theta_{e}\left(i,n+1\right). \end{array} \end{array}$$

Combining Equations (20) and (21), the torque error at (*i, n* + 1) is

(22) $$\begin{array}{l} e_{\tau }\left(i,n+1\right)\\ \begin{array}{c} =\tau \left(i,n+1\right)-\tau_{des}\left(i,n+1\right)\\ =\left(K_{des}-K_{t}\right)\cdot \left[\left(1-K_{t}K_{l}R\right)\cdot \Delta \theta_{e}\left(i,n\right)+\Delta \theta_{e}\left(i,n+1\right)\right] \end{array} \end{array}$$

Assuming that the ankle kinematics change is bounded, i.e.,

$$\|\Delta \theta_{e}\left(i,n\right)\|\leq \epsilon \;\forall n\in R^{1}$$

Then, the error at (*i, n*),

(23) $$\begin{array}{l} \left\|e_{\tau }\left(i,n\right)\|=\|\left(K_{des}-K_{t}\right)\cdot \Delta \theta_{e}\left(i,n\right)\right\|\\ \;\leq \|\left(K_{des}-K_{t}\right)\|\cdot \|\Delta \theta_{e}\left(i,n\right)\|\\ \;=\|\left(K_{des}-K_{t}\right)\|\cdot \epsilon \end{array}$$

The error at (*i, n* + 1) then has

$$\begin{array}{c} e_{\tau }\left(i,n+1\right)\\ \begin{array}{c} =\left(K_{des}-K_{t}\right)\cdot \left[\left(1-K_{t}K_{l}R\right)\cdot \Delta \theta_{e}\left(i,n\right)+\Delta \theta_{e}\left(i,n+1\right)\right]\\ \leq \|K_{des}-K_{t}\|\cdot \left[\|1-K_{t}\cdot K_{l}\cdot R\|+1\right]\cdot \epsilon \end{array} \end{array}$$

It is still in our best interest to assert

$$1-K_{t}\cdot K_{l}\cdot R=0.$$

Therefore, we make the following hypothesis.

**Hypothesis 1**. *There is an optimal iterative learning gain that benefits real-time torque tracking performance of iterative learning in exoskeleton assisted walking under a linear spring-like desired torque profile*:

(24) $$\begin{array}{l} K_{l,opt}=\frac{1}{K_{t}R} \end{array}$$

### 2.4. Experimental Methods

The experiments of this study was conducted not to quantify human reactions but the performance of various torque control conditions. Therefore, only one healthy subject (*N* = 1, female, 32 years, 1.65 m, 56 kg) was involved. In all experiment sessions, the subject walked with a self-paced frequency on a treadmill running at 1.25 m/s while wearing the ankle exoskeleton on the right foot. All experimental protocols were approved by Carnegie Mellon University IRB.

In presentation of all experimental methods and results, this paper uses meter, Newton-meter and degree as the units for distance, torque and angle. The testbed system had an aspect ratio *R* = 2.5 and a gear ratio of *N* = 5. In all experiments, low level control parameters *T* in Equation (6) was set as 50 ms.

#### 2.4.1. Generation of Desired Torque Curves

**Figure 3:**
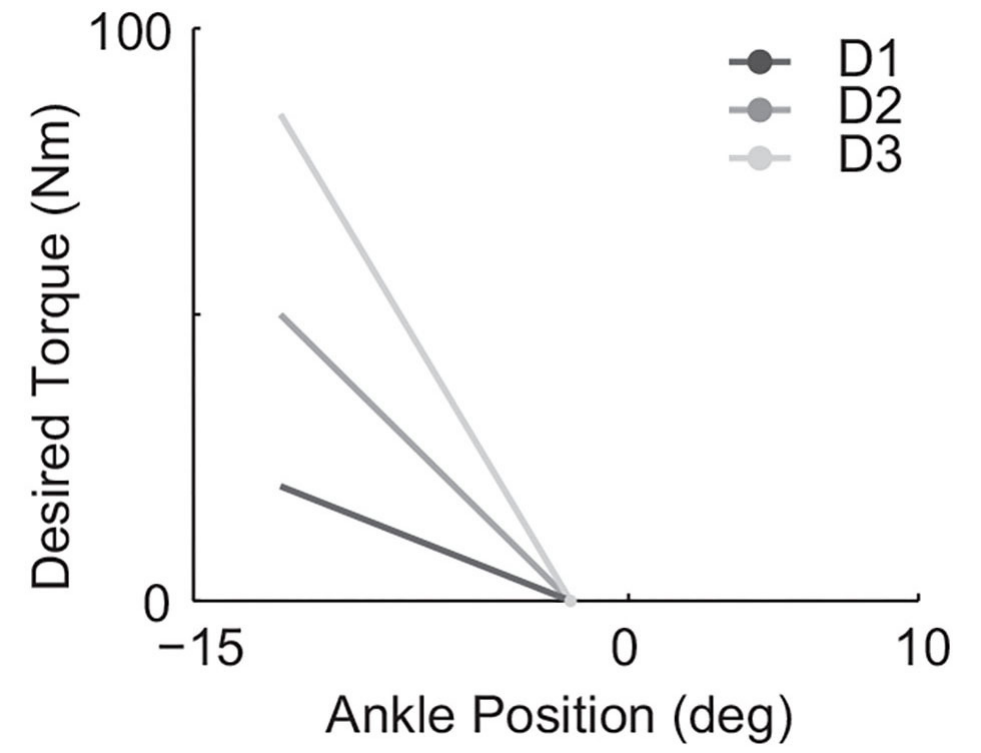
All three tested linear desired torque vs. ankle angle curves used in the form of Equation (7) with θ_*e*,0_ = −2(deg) and *K*_*des*_ values listed in Table 1.

Three different desired quasi-stiffnesses in the form of Equation (7) were implemented to test the hypothesis. In all cases, θ_*e*,0_ = −2(deg), in which θ_*e*_ = 0 was defined by the neutral standing position. The tested desired stiffness *K*_*des*_ spans (Table 1) with a maximum value that is 4.25 times the minimum. The resulting desired torque-ankle-angle relationships are demonstrated in Figure 3.

**Table 1:** List of desired stiffness tested in experiments with assigned ID.

**Desired stiffness ID**	**D1**	**D2**	**D3**
**K_des_** (Nm/deg)	2	5	8.5

#### 2.4.2. Realization of Different Passive Stiffness and Evaluation of Their Values

With every desired stiffness value defined by a torque-angle curve, we tested it in combination with three different passive transmission stiffness values by changing the series spring in the ankle exoskeleton (Figure 1). Two were realized by attaching different compression springs (Diamond Wire Spring, Glenshaw, PA) and the last one was by eliminating the spring from the structure. In that case, the system passive stiffness equalled the stiffness of the rope in Bowden cable.

**Figure 4:**
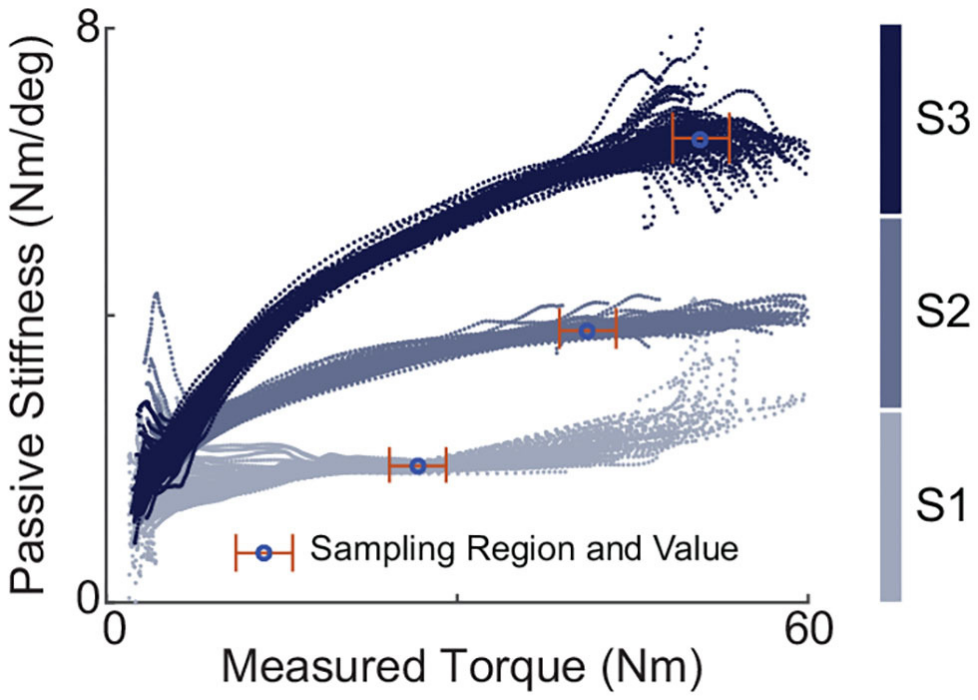
Instantaneous passive stiffness values under passive walking plotted against the measured torques for different spring configurations, one session for each. Each session consisted of one-hundred strides with motor position locked. The stabilized passive stiffness value of one session was defined as the median of the values over a stabilized region. The effective stiffness of the one configuration was defined as the mean of stabilized values of multiple sessions.

The values of effective passive stiffness, *K*_*t*_, with different spring configurations were experimentally evaluated using passive walking where motor position was locked. The process went like this: under each set-up, motor position was fixed where forces began to be generated when the participant stood in a neutral position. Then the participant walked while wearing the exoskeleton on the treadmill for more than one hundred steady strides. Such sessions were done multiple times for each stiffness configurations. For one hundred steady strides of each walking session, the instantaneous passive stiffness value was calculated and plotted against the torque values. Figure 4 shows examples of such plots of passive walking sessions, one session for each spring configuration. Each session produced a stabilized passive stiffness which was defined as the median of the instantaneous stiffness values within a stabilized region. For any passive stiffness set-up, the stabilized region was chosen as a 5.65 Nm torque range, within which the change of the instantaneous stiffness trend averaged over all sessions was minimum. Then, the effective passive stiffness value of a specific spring configuration was defined as the mean of the stabilized passive stiffness values across multiple experimental sessions with this configuration.

The list of springs used and their corresponding properties and their actual values of passive stiffness as calculated with methods discussed above are listed in Table 2. This passive stiffness set spanned a range with a 3.5 times difference between the maximum and minimum values.

**Table 2:** List of passive stiffness values.

**Passive stiffness ID**	**S1**	**S2**	**S3**
Spring part no.	DWC-148M-13	DWC-187M-12	No Spring
Length (m)	0.0635	0.0508	–
Spring rate (N/m ×10^3^)	15.1	50.1	–
Max load (N)	413.7	778.4	–
*K*_*T*_ (Nm/deg)	1.8957	3.6672	5.9365

#### 2.4.3. Experimental Procedures

For each desired stiffness and passive stiffness combination, ten iterative learning gains that span a range with a 20-times difference between the maximum and minimum around the predicted optimal value per Equation (24) (Table 3). Therefore, 3 × 3 × 10 experiment sessions were conducted in total. During each experiment session defined by a unique combination of learning gain, desired stiffness and passive stiffness, the subject walked for at least one hundred strides after stabilization of the learning processes.

**Table 3:** List of iterative learning gain values tested in experiments (deg/Nm).

	**Passive stiffness ID**		
**Desired stiffness ID**	**S1**	**S2**	**S3**
D1	0.0443, 0.0593, 0.0885, 0.1328, 0.1770, 0.2655, 0.3540, 0.5310, 0.7081, 0.8851	0.0354, 0.0443, 0.0593, 0.0885, 0.1328, 0.1770, 0.2655, 0.3540, 0.5310, 0.7081	0.0266, 0.0354, 0.0443, 0.0593, 0.0885, 0.1328, 0.1770, 0.2655, 0.3540, 0.5310
D2	0.0443, 0.0593, 0.0885, 0.1328, 0.1770, 0.2655, 0.3540, 0.5310, 0.7081, 0.8851	0.0354, 0.0443, 0.0593, 0.0885, 0.1328, 0.1770, 0.2655, 0.3540, 0.5310, 0.7081	0.0266, 0.0354, 0.0443, 0.0593, 0.0885, 0.1328, 0.1770, 0.2655, 0.3540, 0.5310
D3	0.0443, 0.0593, 0.0885, 0.1328, 0.1770, 0.2655, 0.3540, 0.5310, 0.7081, 0.8851	0.0354, 0.0443, 0.0593, 0.0885, 0.1328, 0.1770, 0.2655, 0.3540, 0.5310, 0.7081	0.0266, 0.0354, 0.0443, 0.0593, 0.0885, 0.1328, 0.1770, 0.2655, 0.3540, 0.5310
Predicted optimum *K*_*l, opt*_	0.2107	0.1089	0.0674

The theory-predicted optimal iterative learning gain values according to Hypothesis 1 were also listed in the table as a comparison.

**Figure 5:**
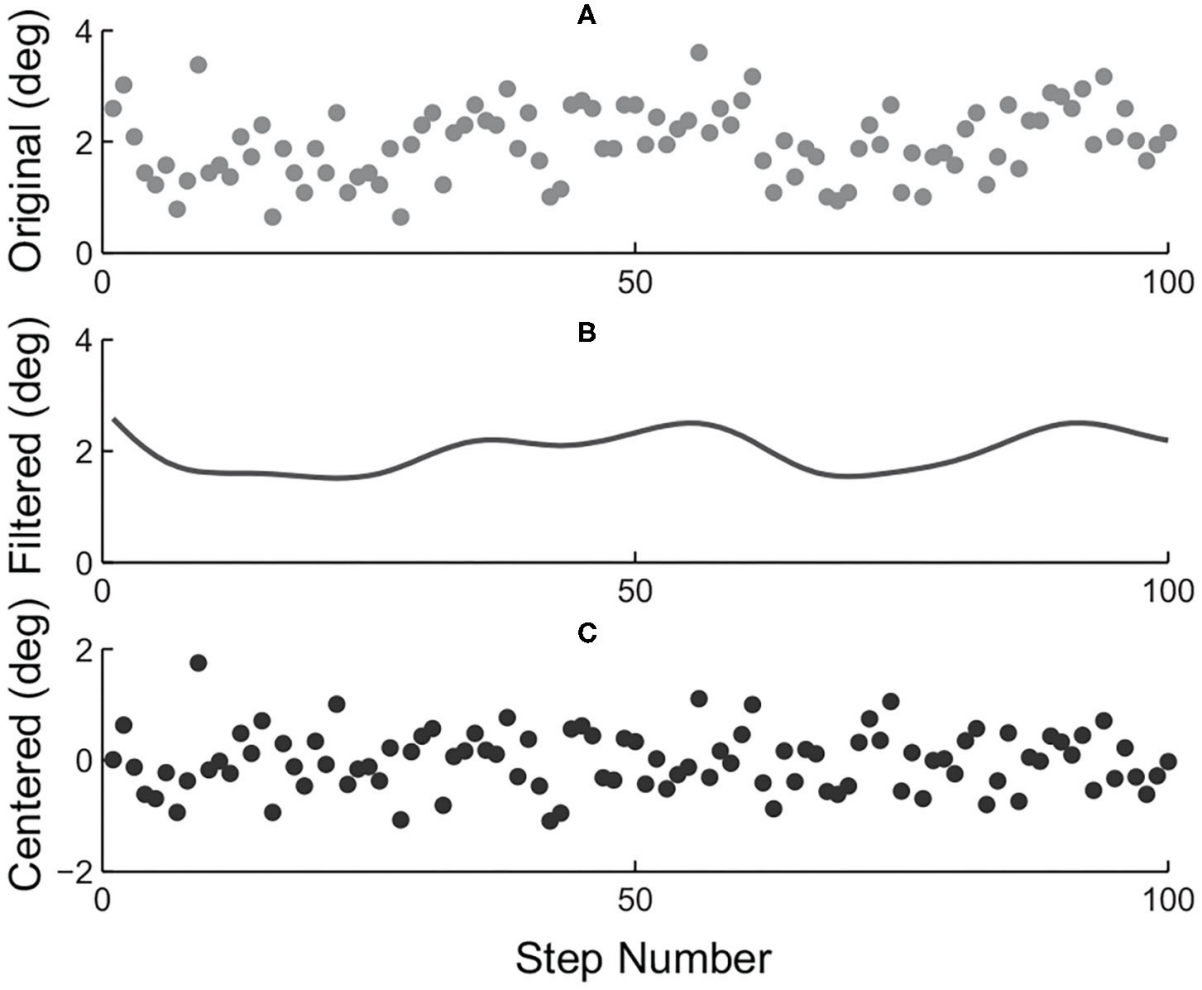
Centering process of index-wise ankle positions. **(A)** Ankle position array of the one hundred strides investigated for an example time index within strides. **(B)** Ankle position array as shown in **(A)** zero-phase filtered with a 1/20 cut-off frequency butter-worth filter. **(C)** Centered ankle position array achieved by subtracting array in **(B)** from that in **(A)**.

Four different indicators of torque tracking performance were calculated for each combination. The first as we call an “absolute error” was defined as the mean of stride-wise root-mean-squared torque errors over the one hundred stable strides. The second one was a “relative error” defined by the absolute error divided by the mean of peak desired torque of the one hundred stable strides. Besides, the normalized instantaneous torque error value of a specific time index within the stride was defined by dividing the original torque error by the standard deviation of a centered 1 × 100 ankle position array of the same time index over all one hundred strides. A centered ankle position array of one particular time index of the stride was defined as the difference between the original array and filtered array as demonstrated in Figure 5. The resulting values were then used to calculate the “normalized absolute error” and the “normalized relative error” in a similar fashion as those of the “absolute error” and “relative error.”

For each unique combination of desired and passive stiffness values, we investigated the relationship between the defined error indices and the corresponding learning gains to test the hypothesis.

## 3. Results

**Figure 6:**
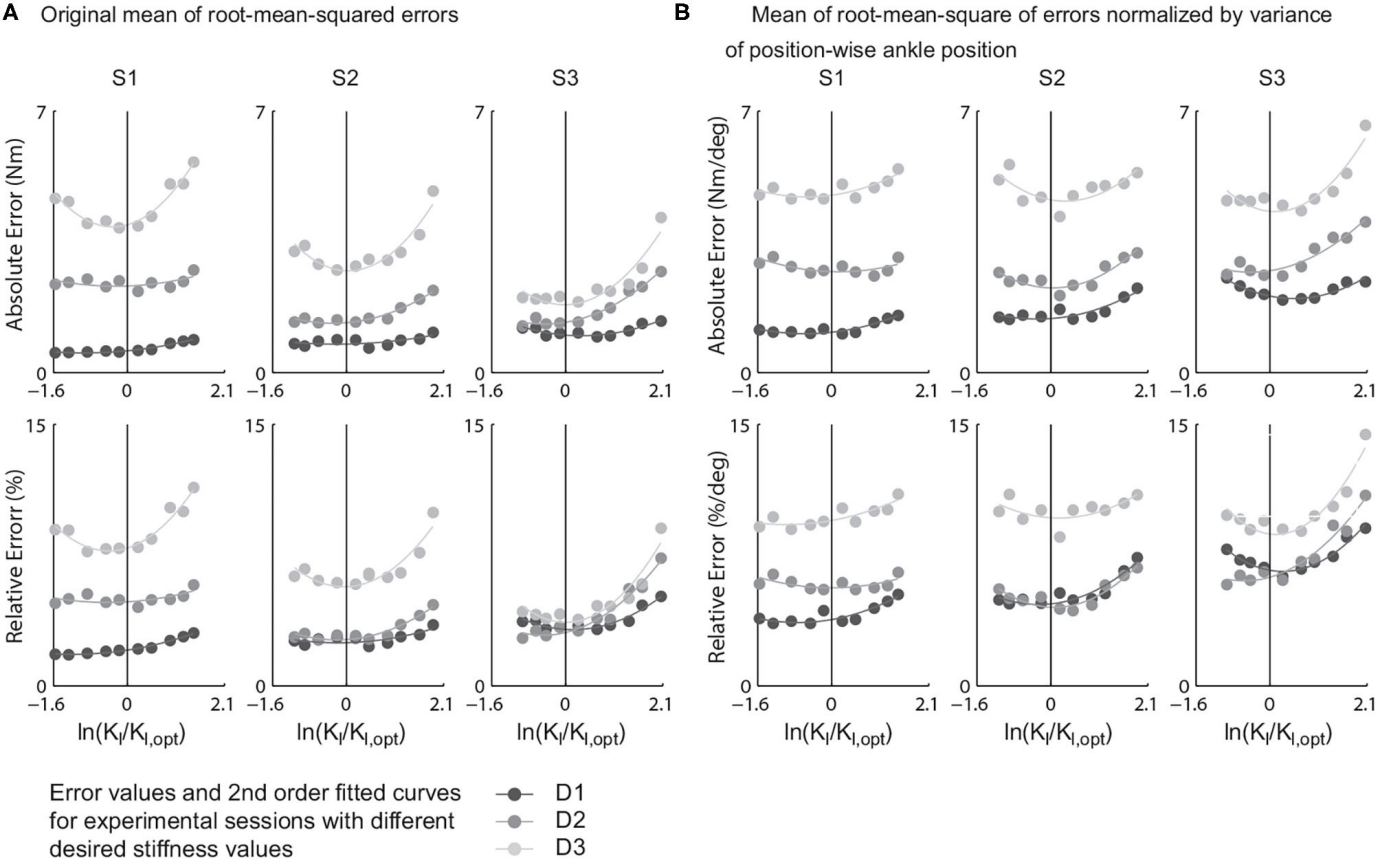
Mean of stride-wise root-mean-squared torque tracking errors of all {gain, desired stiffness, passive stiffness} combinations and the relative errors to their peak desired torques. **(A)** Values computed with raw torque errors. **(B)** Values computed by normalizing raw torque errors by position-wise ankle position variance.

The mean of root-mean-squared torque errors of various learning gains were presented against the ratio of actual learning gains to the theory predicted one in logarithmic scale, i.e., $ln\left(\frac{K_{l}}{K_{l,opt}}\right)$ (Figure 6). For each combination of passive stiffness values and a desired torque curves, the values tracking errors of and $ln\left(\frac{K_{l}}{K_{l,opt}}\right)$ were fitted into a second order polynomial, i.e.,

$$e\left(ln\left(\frac{K_{l}}{K_{l,opt}}\right)\right)=a{\left(ln\left(\frac{K_{l}}{K_{l,opt}}\right)\right)}^{2}+bln\left(\frac{K_{l}}{K_{l,opt}}\right)+c.$$

We defined the *K*_*l*_ value at which the value of the polynomial was minimized as the experimental optimum of iterative learning gain and label it as *K*_*l,opt,exp*_. The $\frac{K_{l,opt,exp}}{K_{l,opt}}$ values of all combinations are listed in Table 4.

**Table 4:** Calculated relative optimal iterative learning gain values *K*_*l,opt,exp*_/*K*_*l,opt*_ through curve-fitting experimental data.

**Stiffness combination (desired, passive)**	**RMS tracking errors**		**Normalized RMS tracking errors**	
	**Absolute**	**Relative**	**Absolute**	**Relative**
(D1, S1)	0.3923	0.3047	0.5667	0.4823
(D2, S1)	0.7779	0.6472	1.1623	0.9884
(D3, S1)	0.7307	0.6726	0.5850	0.3731
(D1, S2)	0.8006	0.8374	0.7524	0.7738
(D2, S2)	0.7472	0.8150	1.0389	1.0767
(D3, S2)	1.0377	0.9533	1.3639	1.1933
(D1, S3)	1.4233	1.1708	1.6885	1.3190
(D2, S3)	0.6286	0.6147	0.6616	0.5767
(D3, S3)	0.9768	1.0023	1.1173	1.1332
Average	0.8350 ± 0.2892	0.7798 ± 0.2547	0.9929 ± 0.3846	0.8796 ± 0.3398

From the results, for all tracking error definitions and all combinations of desired and passive stiffness values, the minimum tracking errors happen in a rather closed neighborhood of *K*_*l*_ = *K*_*l,opt*_, which agreed with **Hypothesis 1**. Furthermore, among all four torque error indices, the absolute error normalized to the standard deviation of centered ankle position trajectories showed experimental optimum closest to theory prediction. The average of experimental optimal gains under nine different combinations of passive and desired stiffness values was 0.9929 ± 0.3846 times the predicted optimum (Table 4).

Besides, the slope between the error and learning gain is rather shallow in the area around the expected optimal gain for all combinations of desired and passive stiffness values. A 100% increase in learning gain only results in a 4.54% increase in normalized torque error on average.

## 4. Discussion

We examined the possibility of optimizing the real-time torque tracking performance of iterative learning control after the stabilization of learning process in series elastic actuator driven walking robots. We made predictions based on theoretical analysis and tested the theory using exoskeleton assisted human walking experiments.

Theoretic analysis suggested that to attenuate the effects of gait variations to minimum using iterative learning feed-forward torque control, the gain should be set as the inverse of the actuator passive stiffness with respect to the motor side. Among the various torque error indices investigated for walking experiments, torque errors normalized by the standard deviation of the centered ankle position array demonstrated strongest agreement with this hypothesis. The experimental optimal iterative learning gain identified was 0.9929 ± 0.3846 times the predicted one, i.e.,

(25) $$\begin{array}{l} K_{l,opt,exp}=\left(0.9929\pm 0.3846\right)\times K_{l,opt} \end{array}$$

These results showed that this optimal learning gain mostly suppressed the torque errors due to the step-to-step variation of human gait after stabilization, but not those due to slow adaptation of human gaits, which agreed with the theoretical analysis and the purpose of this study. In ankle exoskeleton assisted walking experiments and sessions with less stable gait patterns due to participant conditions or environment, this result is expected to be highly useful.

The results also suggested a shallow slope of tracking error increase when the learning gain deviated from the hypothesized optimum. A 100% increase in learning gain only results in a 4.54% increase in normalized torque error on average. This means a rather robust performance of the theory in this study, which is especially meaningful in exoskeletons. The high non-linearity of the series spring and time-varying property of the transmission and interaction subsystems all mean a difficult system identification process and constantly present model-system mismatch. Besides, the fairly flat bottom of the error-vs.-gain curve also partially explained the relatively big standard deviation of the experimental optimum (0.3846 as in Equation 25).

Due to the presence of the non-linear, complex, and time-varying system dynamics and the employment of a highly simplified model, many features were not reflected in the theoretical hypothesis, which led to imperfection in the alignment between theory and experiment results. One issue that contributed was the non-linearity of the passive stiffness coming from the slow stretching of the synthetic rope as demonstrated by Figure 4. Besides, there existed unstructured changes of passive stiffness between loads and trials, but only one single stabilized value was used for one passive stiffness setup. Another complication of the system dynamics not accounted for in theoretical analysis was the highly non-linear, complex, and time-varying static and dynamic frictions in Bowden cable. We also assumed perfect motor position tracking, which was not true in practical cases due to the limitation of motor velocity.

Regardless of the imperfection of system modeling, the torque tracking errors did arrive at a minimum at the neighborhood of the hypothesized optimal iterative learning gain. The shallow slope of changes around the optimum value also suggested a rather relaxed learning gain tuning process. When the iterative learning gain spans a range of [50% 200%] relative to the theoretical optimum, the average increase in torque error is expected to be <5% of that at the optimal gain. Considering a relative torque error of only 2–8% of desired torque at the optimum, a 5% increase on top is rather insignificant and will not harm exoskeleton performance or accuracy of experiments.

This study is the last part of a series of three studies focusing on improved torque tracking for lower-limb exoskeletons during walking. The first one compared a group of prominent controllers used in this type of devices, and identified model-free, integral-control-free feedback combined with feedforward iterative learning as the most effective controller structure, with the potential to reduce torque tracking error to around 1% of peak desired torque (Zhang et al., 2015, 2017a). The second optimized the passive stiffness value of the series elastic actuator of the exoskeleton (Zhang and Collins, 2017). This last study aimed to further improve the torque tracking performance by tuning iterative learning gain in presence of stride-to-stride human gait variations after stabilization of learning. When the combination of feedback control and iterative learning stabilizes during human walking, the real-time control output mainly comes from the learning part (Zhang et al., 2015, 2017a). Just like a traditional PID control, at the end of which the control output was mainly contributed by the integral control part. Therefore, in analysis and experiments of this study, only the iterative learning part was discussed.

A lot of complications, such as frictions and other non-linearities, uncertainties, time-varying dynamics were not discussed or featured in the analysis of this study. The reason was that previous studies (Zhang et al., 2015, 2017a) have shown that in presence of all these complications, a combination of model-free, integration-free control with iterative learning was most effective. The reason was that it is analogous to a traditional PID control, in which tracking, stability and steady-state errors were all dealt with.

Besides, stability of iterative learning was also solved in previous studies (Zhang et al., 2015, 2017a). Errors accumulated and propagating along the way can be suppressed by adding a forgetting factor, error filtering factor and an optional resetting process during human walking.

## 5. Conclusions

This study is the last part of a trilogy of studies on accurate torque tracking for lower-limb exoskeletons during human walking, followed by the identification of an effective control structure (Zhang et al., 2015, 2017a) and the optimization of device series elastic actuator passive stiffness (Zhang and Collins, 2017). It hypothesized the existence and value of an optimal iterative learning gain for real-time torque tracking in ankle exoskeleton during walking with gait variations and validated it with walking experiments. The optimal gain was identified as the inverse of transmission stiffness relative to the motor side. This result further improved exoskeleton torque tracking performance considering limited knowledge of the human and interaction dynamics we can achieve with current technologies. It provided clear guide to iterative learning gain tuning process in exoskeleton systems. Besides, a shallow slope of changes in tracking errors around the neighborhood of the optimal gain suggested a rather robust performance, which is especially meaningful for exoskeleton systems that are complicated, time-varying, and difficult to do system identification. Based on the results of this study, we recommend a iterative learning gain range that is [50% 200%] of the hypothesized optimum $\frac{1}{K_{t}R}$.

## Data Availability Statement

The raw data supporting the conclusions of this article will be made available by the authors, without undue reservation.

## Ethics Statement

The studies involving human participants were reviewed and approved by Carnegie Mellon University IRB. The patients/participants provided their written informed consent to participate in this study.

## Author Contributions

All authors listed have made a substantial, direct and intellectual contribution to the work, and approved it for publication.

## Conflict of Interest

The authors declare that the research was conducted in the absence of any commercial or financial relationships that could be construed as a potential conflict of interest.
